# *Areca palm velarivirus 1* infection caused disassembly of chloroplast and reduction of photosynthesis in areca palm

**DOI:** 10.3389/fmicb.2024.1424489

**Published:** 2024-06-13

**Authors:** Xianmei Cao, Baosen Gao, Jie Lu, Hongxing Wang, Ruibai Zhao, Xi Huang

**Affiliations:** School of Breeding and Multiplication (Sanya Institute of Breeding and Multiplication), Hainan University, Haikou, Hainan, China

**Keywords:** *Areca catechu*, co-evolution, cytopathology, ultrastructure, Yellow Leaf Disease

## Abstract

The expansion of betel palm cultivation is driven by rising demand for betel nut, yet this growth is accompanied by challenges such as decreased agricultural biodiversity and the spread of infectious pathogens. Among these, Yellow Leaf Disease (YLD) emerges as a prominent threat to betel palm plantation. *Areca Palm Velarivirus 1* (APV1) has been identified as a primary causative agent of YLD, precipitating leaf yellowing, stunted growth, and diminished yield. However, the precise mechanisms underlying APV1-induced damage remain elusive. Our study elucidates that APV1 infiltrates chloroplasts, instigating severe damage and consequential reductions in chlorophyll a/b and carotene levels, alongside notable declines in photosynthetic efficiency. Moreover, APV1 infection exerts broad regulatory effects on gene expression, particularly suppressing key genes implicated in chloroplast function and photosynthesis. These disruptions correlate with growth retardation, yield diminishment, and compromised nut quality. Intriguingly, the paradoxical destruction of the host's photosynthetic machinery by APV1 prompts inquiry into its evolutionary rationale, given the virus's dependence on host resources for replication and proliferation. Our findings reveal that APV1-induced leaf yellowing acts as a beacon for transmission vectors, hinting at a nuanced “host-pathogen-vector co-evolutionary” dynamic.

## Introduction

The betel palm (*Areca catechu* L.) is one of the most economically significant crops cultivated in Southeast Asia, with its commercial value being highest in China and India due to its widespread popularity as a chewable product (Khan et al., 2023). In 2020, the total output value of betel nut fruit in Hainan province, China, reached 14.68 billion yuan (2 billion USD), becoming a vital source of income for 2.2 million farmers, accounting for ca. 40% of the agricultural population in Hainan (Khan et al., 2022). Presently, there are 41,800 enterprises related to betel nut processing, employing nearly 300,000 people. The annual output value of China's betel nut industry has reached 60 billion yuan (8.3 billion USD). The increasing betel nut consumption has drew global attention to the health issue of this additive substance (Chen et al., 2023). Additionally, the increasing demand for betel nuts has driven the continuous expansion of betel palm cultivation. Widespread monoculture has led to a decrease in agricultural biodiversity, resulting in an increasing threat from insects and diverse phytopathogens. Among these threats, Yellowing Leaf Disease (YLD) stands out as the most devastating (Johnson et al., 2015; Wang et al., 2020; Khan et al., 2022, 2023). As the name suggests, leaf chlorosis is the most prominent symptom, and in the later stages, the palm's crown size decreases, and “bunchy top” symptoms appear, causing a sharp decline in the yield and quality of betel nut (Yu et al., 2015; Cao et al., 2021; Zhang et al., 2022).

Although YLD has long been associated with phytoplasma (Nayar and Seliskar, 1978; Kanatiwela-de Silva et al., 2015), the causal agent of phytoplasma remains controversial due to the lack of convincing evidence (Purushothama et al., 2007). In recent years, *Areca palm velarivirus 1* (APV1) was identified in YLD samples through RNA sequencing (Yu et al., 2015; Cao et al., 2021; Zhang et al., 2022). Field investigations have shown a strong association between APV1 and YLD (Wang et al., 2020). Furthermore, APV1 can be transmitted by both *Pseudococcus cryptus* and *Ferrisia virgata*, causing typical YLD symptoms in betel palm seedlings, suggesting that APV1 might be the causal agent of YLD (Zhang et al., 2022; Zhao et al., 2024). APV1 has been identified as a member of the genus *Velarivirus* in the family *Closteroviridae* according to phylogenetic analysis and genome structure (Yu et al., 2015; Wang et al., 2020).

Viruses in the *Closteroviridae* family have non-enveloped filamentous particles ranging from 650 to 2200 nm in length and 12 nm in diameter, with mono-, bi-, or tripartite positive-sense RNA genomes ranging from 13 to 19 kb (Karasev, 2000; Martelli, 2019). According to the 2020 ICTV virus taxonomy profile, the *Closteroviridae* family consists of four genera: *Closterovirus, Ampelovirus, Crinivirus*, and *Velarivirus* (Martelli, 2019; Fuchs et al., 2020). Recently, three new genera (*Bluvavirus, Menthavirus*, and *Olivavirus*) were added to the *Closteroviridae* family (https://ictv.global/taxonomy). As the names of these viruses suggest, many species of closterovirus, such as *Carrot yellow leaf virus, Wheat yellow leaf virus, Cucurbit yellow stunting disorder virus* (Nagendran et al., 2023), and *Sweet potato chlorotic stunt virus* (Zhao et al., 2022), as well as *Tomato chlorosis virus* (Liu et al., 2022), cause yellowing symptoms. However, there is limited articles published about the interactions between closterovirus and chloroplasts, and further investigation is needed to understand the mechanism underlying the leaf yellowing symptom.

Damage to the chloroplast and modifications to photosynthesis are common and conserved strategies employed by plant viruses to establish an optimal niche for infection (Zhao et al., 2016). Understanding the interactions between chloroplasts and viruses is crucial for unraveling the mode of infection and viral pathogenicity (Bhattacharyya and Chakraborty, 2018). APV1 infection causes leaf yellowing, growth retardation, yield loss, decreased nut quality and even tree death. However, the precise mechanisms underlying remain elusive, the aim of the work is to investigate the APV1-induced damage of ultrastructure and related cytopathology. The data presented in this work indicate that APV1 infection induces chloroplast disassembly, degradation of chlorophyll and carotene, and decreased photosynthesis efficiency, which may contribute to the loss of yield and decreased quality of betel nut products.

## Methods and materials

### Plants, mealybugs, and inoculation

Mealybugs (*F. virgata*) were originally isolated from betel palm and were reared on pumpkin fruit in nylon net cages (75 × 75 × 75 cm). One-year old betel palm seedlings and mealybugs were cultivated in chamber as previous report (Zhang et al., 2022). First instars of *F. virgata* were transferred from pumpkin fruit to APV1 infected plants for 48-h acquisition access period (AAP) then further transferred onto test seedlings for 7 days inoculation access period (IAP). The seedlings were sprayed with insecticide to kill the mealybugs after inoculation. A mock inoculation was performed using non-viruliferous mealybugs. The inoculated seedlings were detected by RT-PCR using APV1 specific primers 2 months after inoculation.

### Host choosing experiment

Three groups of betel palm seedlings were chosen for host choosing experiment, including APV1 positive seedlings with yellowing symptom (APV1+ yellow), APV1 positive seedling without yellowing symptom (APV1+ green), and the healthy seedlings (healthy control). After 4 h starvation, 100 adult *F. virgate* mealybugs were released from the cage laying between the two types of *A. catechu* seedlings, the number of mealybugs freely distributed onto each type of seedlings was calculated at 6 h after releasing, respectively.

### Western-blot analysis

Leaves from APV1 infected plants and control plants were collected and ground in liquid nitrogen for Western-blot analysis. Total proteins were isolated and separated by SDS-PAGE. The protein was transferred from gel onto PVDF membrane using a Mini Trans-Blot Electrophoretic Transfer system (Bio-Rad, #1703930). APV1 was detected using a mouse anti-APV1 CP monoclonal antibody (1:5,000 dilution) and horseradish peroxidase-conjugated goat anti-mouse IgG (1:2,000 dilution) as the secondary antibody (Solarbio, catalog no. SE131). The blot was visualized using a chemiluminescence film (Thermo Fisher, catalog no. 34577).

### Differential gene expression analysis

Leaf samples from the APV1-inoculated and mock-inoculated betel palm seedlings were collected for RNA-Seq and *de novo* assembly were conducted as previously described (Wang et al., 2020). All differential expressed genes (DEGs) were subjected to gene ontology (GO) and KEGG enrichment analysis using GOseq and KOBAS software, respectively (Li et al., 2020). qRT-PCR was carried out as previously described (Livak and Schmittgen, 2001; Zhang et al., 2022). Briefly, total RNA was purified from leave samples using a plant RNA extraction kit (Tiangen Biotech, Beijing, China), and cDNA was synthesized with a one-step reverse transcription kit (Thermo Fisher Scientific, Waltham, MA, USA). *Actin* gene was chosen for the normalization. The primer sequences used in this experiment are listed in Supplementary Table S1.

### Pigment measurement

The penultimate fully unfolded leaves of YLD betel palm and healthy betel palm were collected, and 0.2 g of each leaf sample was cut into small pieces. These pieces were then homogenized in 25 ml of 95% ethanol and kept in darkness for 24 h. The supernatants were analyzed at 470, 649, and 665 nm, respectively. Chlorophyll was calculated according to Arnon's equation. The grand means of the values were subjected to analysis of variance (ANOVA) by SPSS version 19.0 (Li et al., 2020).

### Photosynthesis measurement

The penultimate fully expanded leaves of the YLD betel palm and healthy betel palm were measured using a portable open gas exchange system with a 3 × 3 cm leaf chamber (LI6800-12A, Li-COR Inc., Lincoln, NE, USA). Measurements were conducted between 9:00 and 11:00 in the morning on clear days with natural fluctuations in air temperature and vapor pressure deficit. The temperature in the climate chamber was set at 28°C and 55% relative humidity. The CO_2_ level was ~400 μmol mol^−1^. Each sample was measured five times (Tseliou et al., 2021).

### Chlorophyll fluorescence measurements

Chlorophyll fluorescence of the YLD betel palm and healthy betel palm were analyzed using a MINI-PAM-II fluorometer (Walz, Germany). The analyses were repeated three times. Induction curves were performed using a MINI-PAM-II fluorometer following the manufacturer's protocol. The fluorescence parameters of the light response curve and induction curve were converted using WinControl-3 software (Walz, Germany), and the graphs were drawn using Excel software (Ivanov and Bernards, 2016).

### Transmission electron microscopy

Transmission electron microscopy (TEM) was conducted as previously described (Jin et al., 2018). Ultrathin sections were cut to 70–90 nm thickness using a Leica EM UC7 ultramicrotome. The sections were sequentially stained with uranyl acetate for 20 min and Reynolds' lead citrate for 5 min. The sections were then viewed with a Hitachi H-7650 or a JEM-1230 transmission electron microscope operated at 80 kV.

### Immune electron microscopy

Immune Electron Microscopy (IEM) was conducted as previously report (Folimonova et al., 2008). The leaf samples were fixed using phosphate buffer containing 1.8% paraformaldehyde and 0.25% glutaraldehyde, and imbedded in LR White resin (London Resin Company, Hampshire, United Kingdom). Immunogold labeling of ultrathin sections (70- to 80-nm) was performed using a purified mouse monoclonal antibody against APV1-CP (concentration: 2 mg/ml, diluted 1:500) as the primary antibody, followed by incubation with goat anti-mouse 12-nm gold conjugate (diluted 1:25; Sigma). The specimens were meticulously observed using a HT7800/HT7700 transmission electron microscope (HITACHI, Japan).

## Results

### Impact of APV1 infection on betel palm leaf pigments

**Figure 1:**
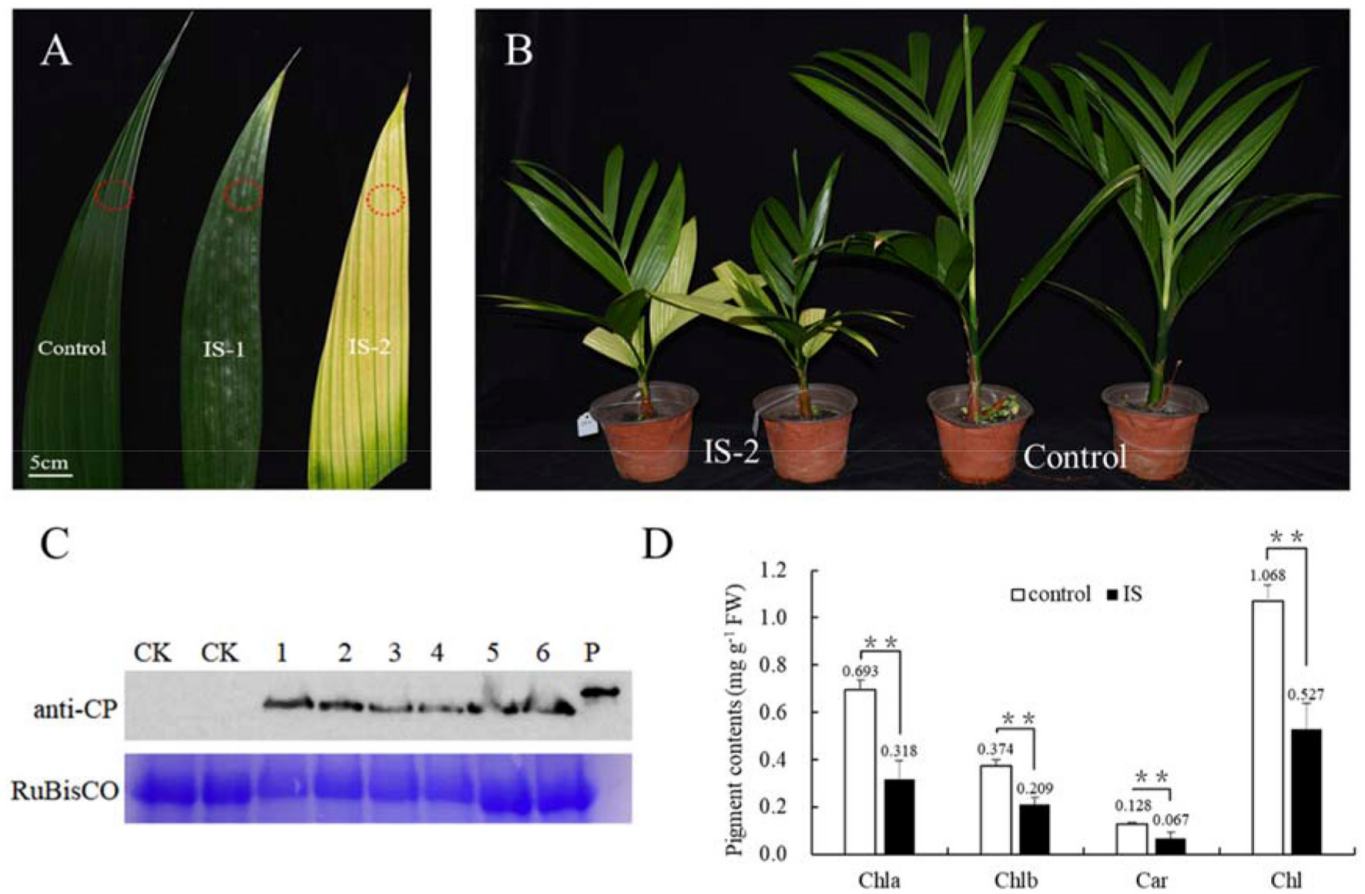
APV1 infection caused yellowing symptom and decreased levels of pigments. **(A)** Leaf yellowing symptoms in infected sample at 60 days (IS-1) and 180 days post inoculation (IS-2) after APV1 inoculation by *F. virgate*. Red rings indicated the positions of collected samples for tissue sections. **(B)** Seedlings symptoms of areca palm at 180 dpi (left) and mock control (right); **(C)** Western-blot indicated the APV1 accumulation after APV1 inoculation using monoclonal antibody against APV1-CP. 1-3: three samples of IS-1, 4-6: three samples of IS-2, CK: mock inoculation, P: His-CP fusion protein isolated from prokaryotic expression in *E. coli*; **(D)** Pigment content in APV1-infected sample (IS-2) or healthy (control) *A. catechu* seedlings. Chla, chlorophyll a; Chlb, chlorophyll b; Car, carotenoid; Chl, chlorophyll a + chlorophyll b. Data are the averages from three independent experiments. The small bars represent standard deviation. Asterisks indicate significant differences between IS and control (**P < 0.01).

To investigate the mechanism underlying the yellowing symptoms induced by APV1 in betel palm, Betel palm seedlings were inoculated with through APV1 through *F. virgate* mealybugs. At 60 days post inoculation (IS-1), leaf yellowing symptoms initially appeared at the leaflet tips. Over time, the yellowing gradually extended toward the petiole, resulting in most of the leaves turning yellow, while the midribs remained green, forming a distinct green-yellow border at 180 days post-inoculation (IS-2) (Figure 1A). This observation aligns with previous reports (Wang et al., 2020; Zhang et al., 2022). In addition to leaf yellowing, APV1-infected seedlings exhibited significant growth retardation compared to the control (Figure 1B; Supplementary Figure S1). Western blot analysis using a monoclonal antibody against APV1-CP revealed the accumulation of APV1 in the inoculated betel palm seedlings (Figure 1C). Measurement results indicated a significant decrease in photosynthesis-related pigments. The contents of chlorophyll a, chlorophyll b, and carotenoids, decreased from 0.693, 0.374, and 0.128 mg/g in control sample to 0.318, 0.209, and 0.067 mg/g in APV1-infected samples, respectively (Figure 1D).

### Effects of APV1 infection on photosynthesis efficiency

**Figure 2:**
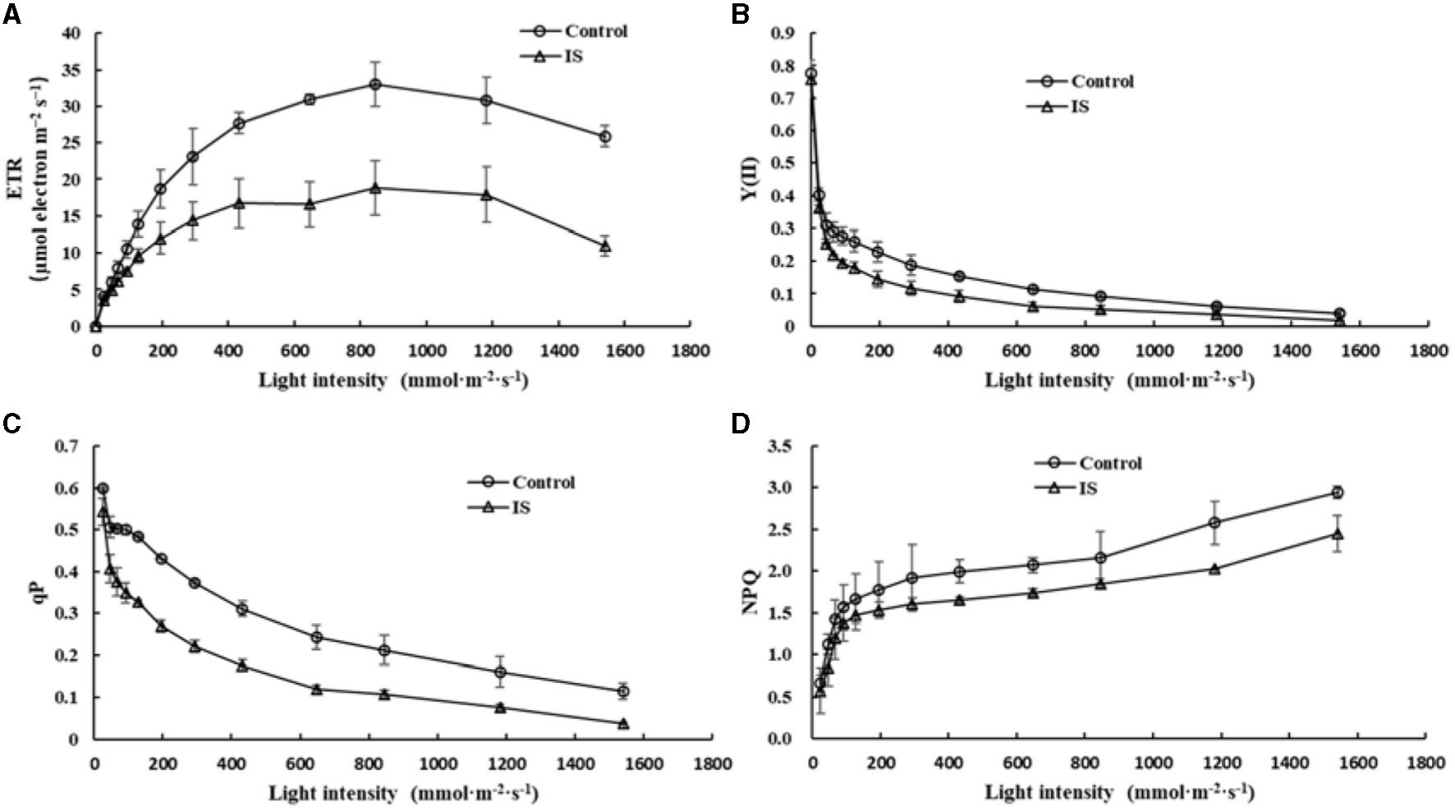
Photosynthesis efficiency affected by APV1 infection. Change of photosynthetic electron transport rate (ETR, **A**), actual photochemical efficiency in photosystem II (Y_II_, **B**), photochemical quenching (qP, **C**), and non-photochemical quenching (NPQ, **D**) were recorded in APV1-infected (IS-2) sample or healthy (control) of areca palm. Data are the averages from three independent experiments.

**Figure 3:**
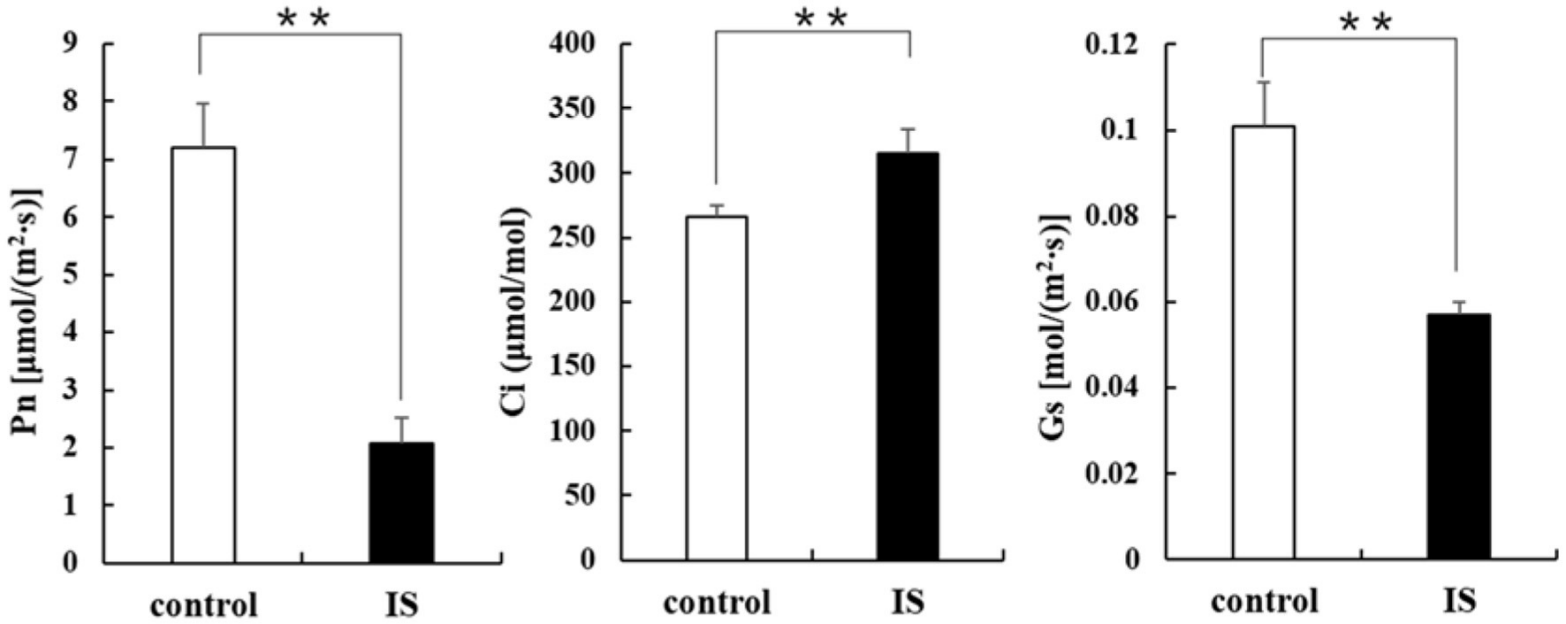
Photosynthesis related parameters affected by APV1 infection. Net photosynthetic rate (Pn), stomatal conductance (Gs), and intercellular carbon dioxide concentration (Ci) were determined in APV1 infected sample (IS-2) of *A. catechu* seedling and mock inoculation (control). Data are the averages from three independent experiments. The small bars represent standard deviation. Asterisks indicate significant differences between IS and control with (**P < 0.01).

Chlorophyll fluorescence can serve as an indicator of plant photosynthesis efficiency and can be used to detect early-stage biotic and abiotic stresses (Moustaka and Moustakas, 2023). Measurement results showed that four crucial indicators of chlorophyll fluorescence, e.g., photosynthetic electron transport rate (ETR), actual photochemical efficiency in photosystem II (Y_II_), photochemical quenching (qP), and non-photochemical quenching (NPQ), were significantly lower in APV1-infected samples (IS-2) than that in the mock control (Figure 2). Furthermore, under conditions of saturated light intensity (1,000 μmol m^−2^ s^−1^), the net photosynthetic rate (Pn) and stomatal conductance (Gs) were significantly lower in APV1-infected samples (IS-2), while the intercellular CO_2_ concentration (Ci) was higher than that of the control (Figure 3). A higher Ci value indicates lower photosynthesis efficiency (Tominaga et al., 2018). In summary, APV1 infection reduced chlorophyll pigmentation, resulted in fluctuation of chlorophyll fluorescence, and led to decreased photosystem efficiency.

### Impact of APV1 infection on chloroplast structure

**Figure 4:**
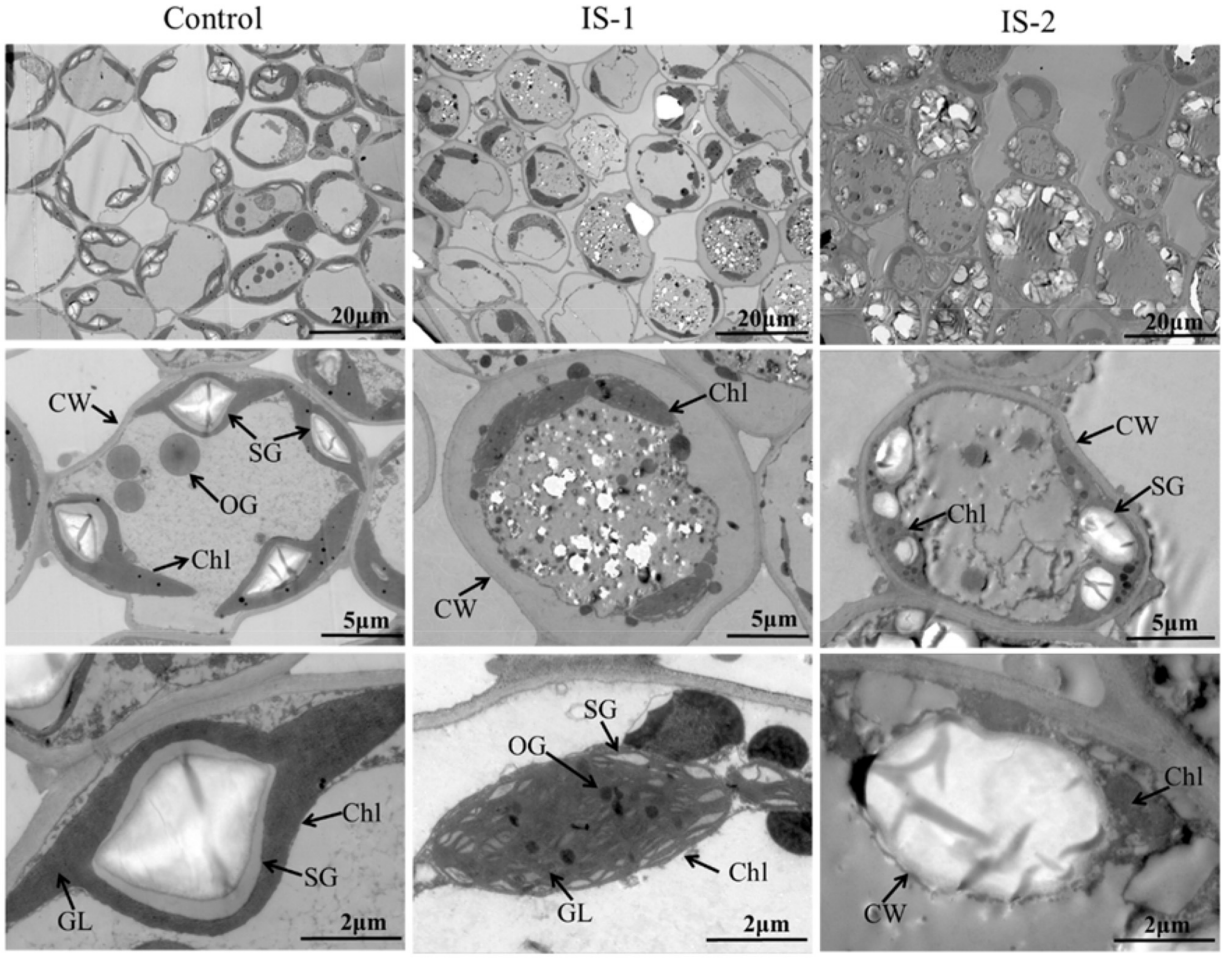
Leaf tissue sections of areca palm were observed under transmission electron microscopy (TEM). Mock control, infected leaf sample at 60 days (IS-1) and 180 days (IS-2) after APV1 inoculation were collected for tissue sections and observed under transmission electron microscopy (TEM). Sampling position for the tissue sections were indicated with red ring in Figure 1A. CW, cell wall; Chl, chloroplast; OG, osmiophilic granule; SG, starch grains; GL, grana lamella are indicated.

**Figure 5:**
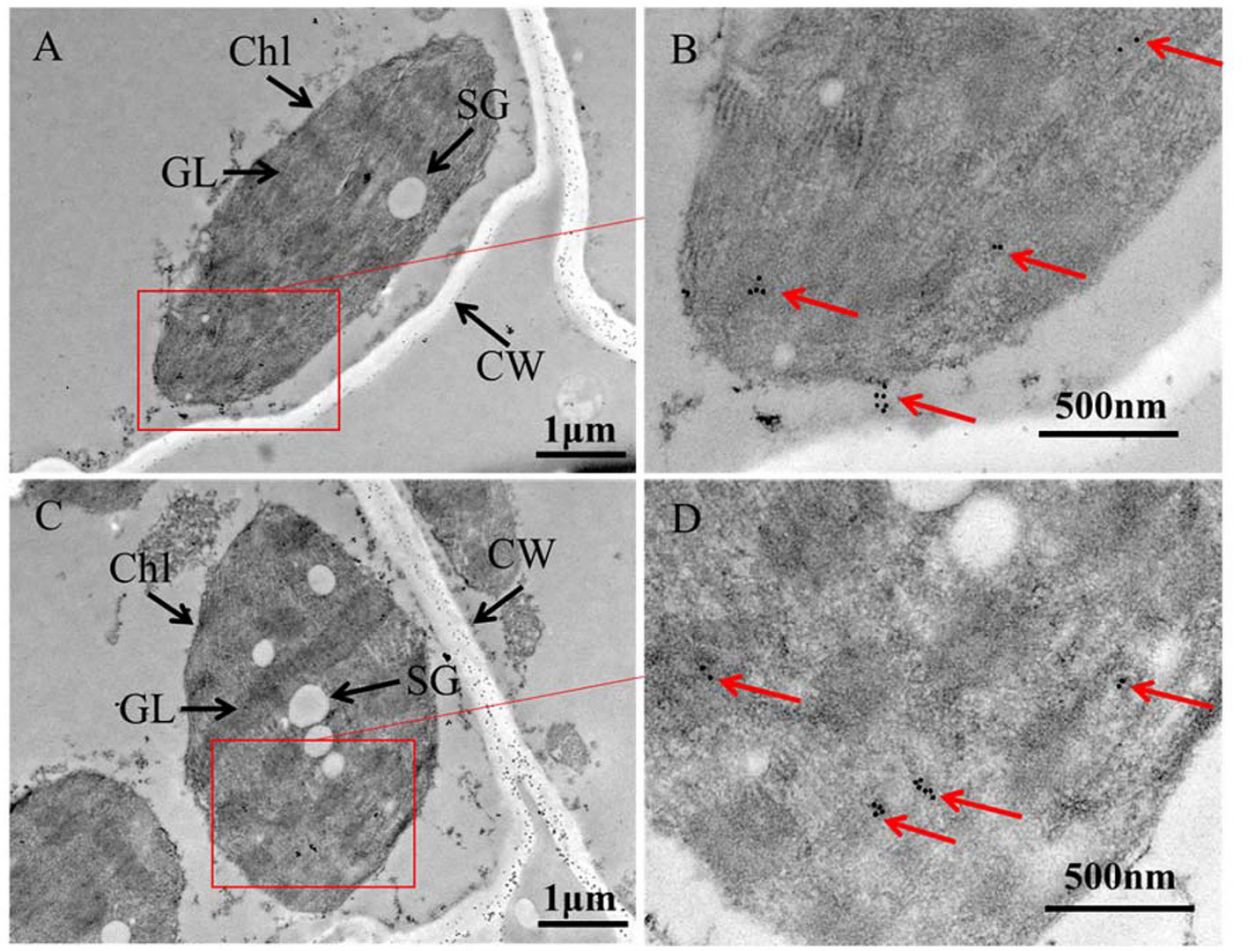
Immune Electron Microscopy (IEM) showing APV1 in chloroplasts of *A. catechu* leave. **(A, C)** Groups of chloroplasts at lower magnification. **(B, D)** Areas from the cells shown in panel at higher magnification. APV1-specific monoclonal antibody was used as primary antibodies and secondary antibodies conjugated with gold particles. CW, cell wall; Chl, chloroplast; SG, starch grains; GL, grana lamella are indicated.

To investigate the causes of yellowing symptoms, reduced chlorophyll, and decreased photosynthesis efficiency induced by APV1, leaves from APV1-infected and healthy control samples were collected for tissue sectioning and transmission electron microscopy (TEM) observation. In healthy control cells, chloroplasts were oval in shape and evenly distributed, with starch grains (SG) surrounded by grana lamellae (GL). In the early stages of virus infection (IS-1), the chloroplast membrane separated from the cell wall (CW), and the size and number of osmiophilic granules (OG) reduced. The GL became less organized, and SG were separated from each other, with many unknown particles and white holes appearing in the cytoplasm. In the late stage (IS-2), chloroplasts were nearly completely degraded, with a sharp reduction in size, and large starch grains (SG) accumulating in the cytoplasm (Figure 4). These findings confirm that APV1 infection severely damages the chloroplast structure and other cytoplasmic organelles in leaf cells. In this work, immune electron microscopy (IEM) was used to examine the distribution of APV1 in leaf. IEM revealed that APV1 mainly localized in chloroplast and occasionally in cytoplasm, confirming that APV1 has invaded chloroplast and caused degradation of chloroplast (Figure 5).

### Gene expression analysis in response to APV1 infection

**Figure 6:**
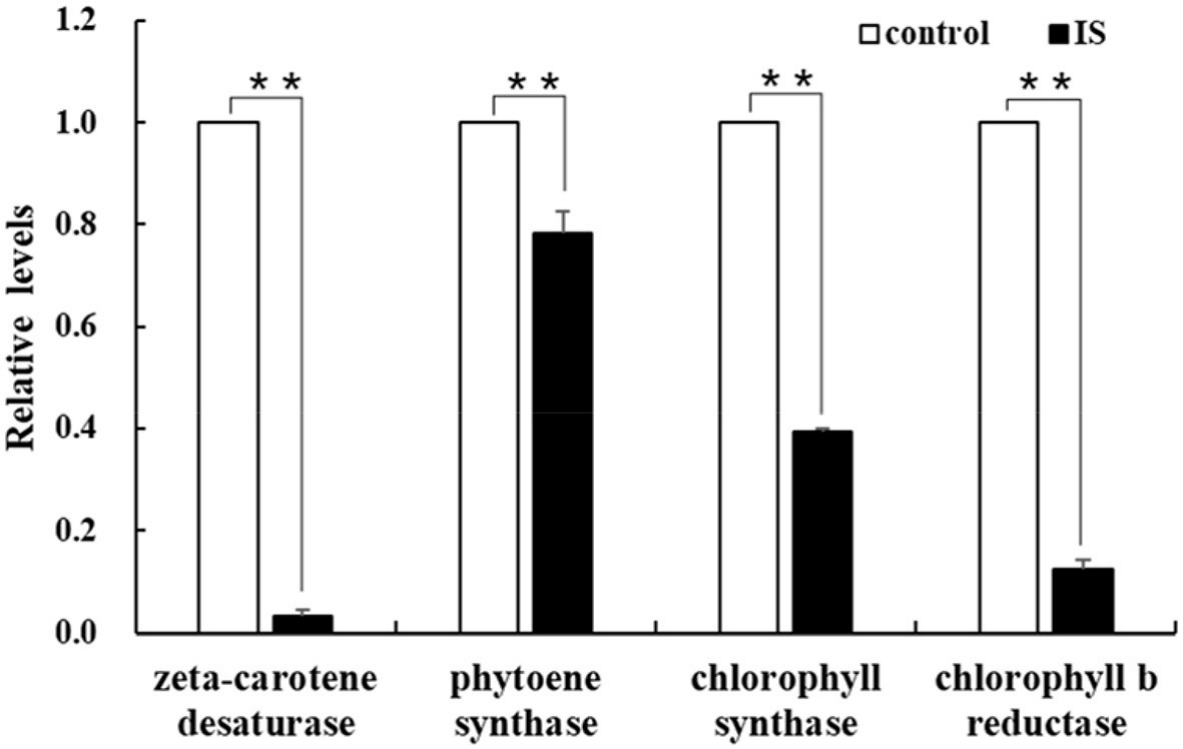
Verification of the differentially expressed genes (DEGs). The expression of chlorophyll and carotene biosynthesis-related genes were analyzed by qRT-PCR. Three independent repetitions have been performed. Statistical data were analyzed by paired sample *t*-test using SPSS software. Asterisk indicates significant difference (**P < 0.01).

To identify genes regulated by APV1 infection, we conducted RNA-seq and analyzed differentially expressed genes (DEGs) between APV1-infected samples and healthy controls. A total of 166,590 unigenes were obtained through *de novo* assembly from the clean RNA-seq reads. DEG analysis revealed 12,553 up-regulated DEGs and 5,698 down-regulated DEGs in response to APV1 infection (Supplementary Figure S2). Gene ontology (GO) annotation enrichment showed that DEGs were distributed across 45 GO terms, with the most DEGs associated with terms such as Binding, Protein binding, and Intracellular organelle (Supplementary Figure S3). Notably, key genes involved in chlorophyll biosynthesis, such as *chlorophyll synthase* (*CS*) and *chlorophyll b reductase* (*CbR*), as well as genes related to carotene metabolism, such as *zeta-carotene desaturase* (*zCD*) and *phytoene synthase* (*PS*), were down-regulated in APV1-infected samples (Supplementary Figure S4). This was further confirmed by qRT-PCR, with the expression of *zCD, PS, CS* and *CbR* significantly down-regulated by APV1 infection (Figure 6). These findings suggest that APV1 infection has a global impact on gene expression patterns, suppressing the expression of key genes involved in chloroplast and photosynthesis, which were nuclear-encoded.

### Yellowing symptoms as attractants for APV1 transmission vectors

**Figure 7:**
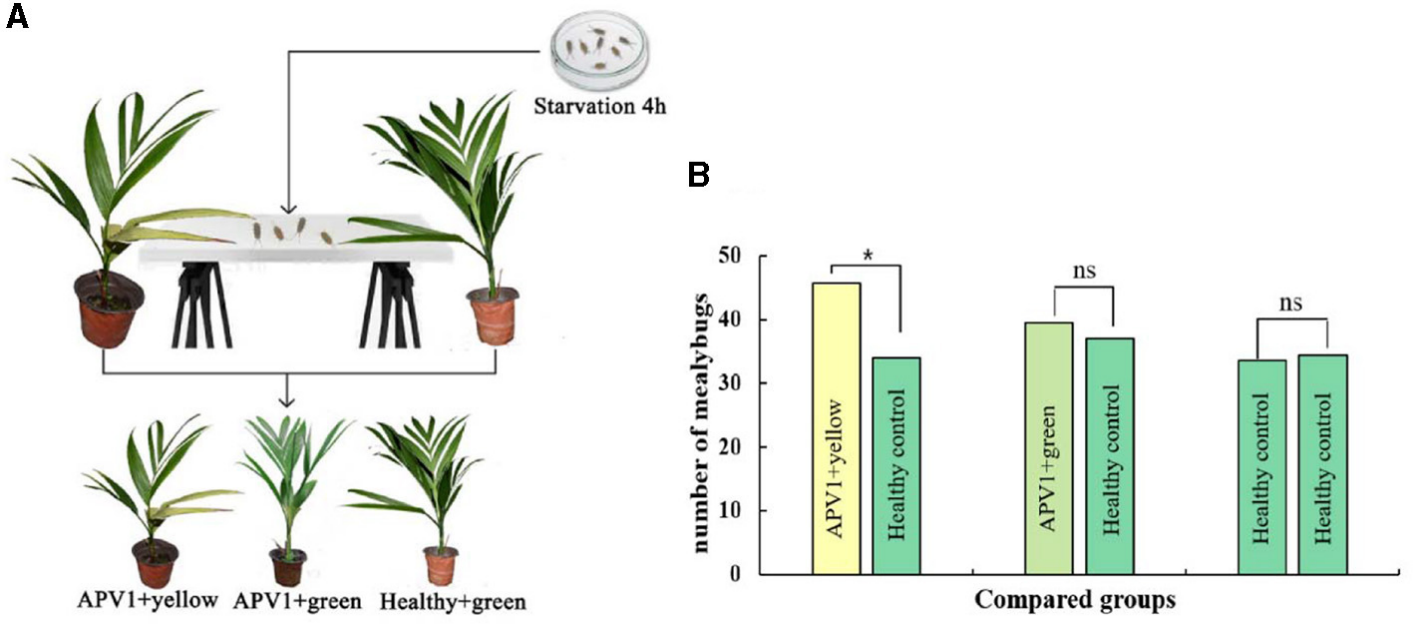
Leaf yellowing symptom is an attractant for *Ferrisia virgate*. **(A)** Schematic diagram for host choosing experiment; **(B)** Three combinations, e.g., APV1+ yellow vs. APV1+ green, APV1+green vs. healthy control, and healthy control vs. healthy control, were compared, respectively. Four independent repetitions have been performed. Statistical data were analyzed by paired sample *t*-test using SPSS software. Asterisk indicates significant difference (*P < 0.05). ns, no significant difference.

To investigate whether yellowing symptoms might attract transmission vectors for the plant virus, the APV1 vector *F. virgate* was used in host-choosing experiments among three types of betel palm seedlings. As expected, there was no significant difference in mealybug distribution between APV1-positive seedlings without yellowing symptoms and healthy seedlings (*t* = 0.458, *P* > 0.05), or between two groups of healthy seedlings (*t* = 0.266, *P* > 0.05). However, a significant difference was observed between APV1-positive seedlings with yellowing symptoms and healthy seedlings (*t* = 3.800, *P* < 0.05), with more mealybugs choosing the seedlings displaying yellowing symptoms as hosts (Figure 7). These results support the hypothesis that yellowing symptoms act as attractants for APV1 transmission vectors.

## Discussion

The chloroplast is a vital organelle in plants responsible for photosynthesis and the biosynthesis of essential biochemical components. Chloroplasts also play a crucial role in defense responses, triggering immune responses against viral pathogens. Paradoxically, chloroplasts are often prime targets for viruses during infections (Zhao et al., 2016; Bhattacharyya and Chakraborty, 2018). Understanding the interactions between chloroplasts and viruses is crucial for unraveling the viral pathogenicity (Bhattacharyya and Chakraborty, 2018). Viruses from the *Closteroviridae* family, such as *Citrus tristeza virus* (CTV) and *Grapevine leafroll-associated viruses* (GLRaVs), have been extensively studied (Karasev, 2000; Albiach-Marti et al., 2010; Dawson et al., 2013, 2015; Naidu et al., 2015). However, the interaction between closteroviruses and chloroplasts has been relatively unexplored. Previous studies showed that citrus tristeza virus (CTV) accumulated preferentially in the phloem parenchyma and to a lesser degree in companion cells and occasionally in sieve elements (Zhou et al., 2002; Folimonova et al., 2008). Ultrastructural analysis also revealed that *Beet yellows closterovirus* (BYV) preferentially accumulated phloem and occasionally in leaf mesophyll cells (Medina et al., 1999). Data from this study demonstrated that APV1 invades chloroplasts and causes the disassembly of chloroplasts and extensive cell structure damage (Figures 4, 5). Additionally, APV1 globally regulated chloroplast photosynthesis-related genes (CPRGs) (Supplementary Figure S4, Figure 6) and reduced the concentrations of chlorophyll a/b, carotene, and photosynthetic efficiency (Figures 2–4). These findings confirm for the first time a central role for chloroplasts in the APV1-plant interaction. Virus infections can cause various degrees of damage to chloroplast structure and lead to malformations, including a reduction in chloroplast numbers (Zechmann et al., 2003; Zhao et al., 2016; Lei et al., 2017; DeBlasio et al., 2018), atypical chloroplast appearances, swollen chloroplasts (Lehto et al., 2003), changes in chloroplast content (Li et al., 2006), decreased grana stack formation, increased starch granule size and number, irregular outmembrane structures (Cowan et al., 2012), and chloroplast disruption (Zhao et al., 2016). APV1 infection completely disrupted the chloroplast structure, resulting in the reduction of chlorophyll a/b, which was exhibited by leaf yellowing symptom. APV1-induced chloroplast disassembly and reduced photosynthesis efficiency should account for such as the growth retardation, yield loss, and decreased nut product quality. This finding points out a direction for future research of YLD.

To fully understand the interaction between APV1 and betel palm, the interaction between the APV1 and chloroplasts should be further investigated. The interaction between chloroplasts and viruses is highly complex and involves the identification of virus-host-interacting proteins (Li et al., 2016; Zhao et al., 2016; Bhattacharyya and Chakraborty, 2018). A preliminary yeast-two-hybrid result showed that APV1-CP interacted with betel palm small heat shock protein (sHSP), a chaperone protein that involved in protein folding (unpublished data), indicating that APV1 might affect chloroplast through the unfolded protein response (Fu et al., 2018; Li et al., 2021). However, this hypothesis requires further investigation. Closing the knowledge gaps in the APV1-chloroplast interaction will aid in utilizing chloroplast-based antiviral resistance and developing management technologies to prevent betel palm chloroplast from APV1 damage.

Finally, plant viruses are obligate biotrophic pathogens reliant on the plant's apparatus and photosynthetic products for replication and proliferation. However, virus infections often result in chloroplast disruption and leaf yellowing symptoms. This raises the question of how infected viruses benefit from these symptoms. Host-preference experiments carried out in this work demonstrated that APV1-induced leaf yellowing symptoms act as an attractant for the APV1 vector, *F. virgate* (Figure 7), suggesting a complex “host-pathogen-vector co-evolution”.

## Conclusion

YLD has a devastating effect on betel palm, causing leaf yellowing, growth retardation, yield loss, decreased nut quality and even death. However, the mechanism underlying is so far rarely reported. Study of cytopathology and ultrastructure of APV1 infected betel palm unraveled that APV1 invaded chloroplasts and caused serious damage of the chloroplasts, resulting reduction of pigments and photosynthesis efficiency. APV1 globally impacted on gene expression patterns and suppressed the expression of nuclear-encoded genes involved in chloroplast and photosynthesis. APV1 induced reduction of photosynthesis efficiency contributed to yield loss and reduced quality of betel nut. Leaf yellowing symptom act as an attractant for *F. virgata*, the APV1 vector, demonstrating a co-evolution.

## Data availability statement

Sequence Read Archive (SRA) raw data are available through the central BioProject database at NCBI under project accession PRJNA1120749 and BioSamples accession SAMN41706791.

## Author contributions

XC: Conceptualization, Formal analysis, Investigation, Methodology, Validation, Writing – original draft, Writing – review & editing. BG: Formal analysis, Investigation, Methodology, Writing – original draft, Writing – review & editing. JL: Formal analysis, Investigation, Methodology, Writing – original draft, Writing – review & editing. HW: Project administration, Supervision, Writing – original draft, Writing – review & editing. RZ: Conceptualization, Project administration, Supervision, Writing – original draft, Writing – review & editing. XH: Funding acquisition, Project administration, Supervision, Validation, Writing – original draft, Writing – review & editing.
