# Supplementary material for: Areca palm velarivirus 1 infection caused disassembly of chloroplast and reduction of photosynthesis in areca palm
Source: Front Microbiol. 2024 Jun 13;15:1424489. doi: 10.3389/fmicb.2024.1424489 (PMC11208678; doi:10.3389/fmicb.2024.1424489)
Supplement: Supplementary file 1 [file Data_Sheet_1.docx]

**Supplementary materials**

**
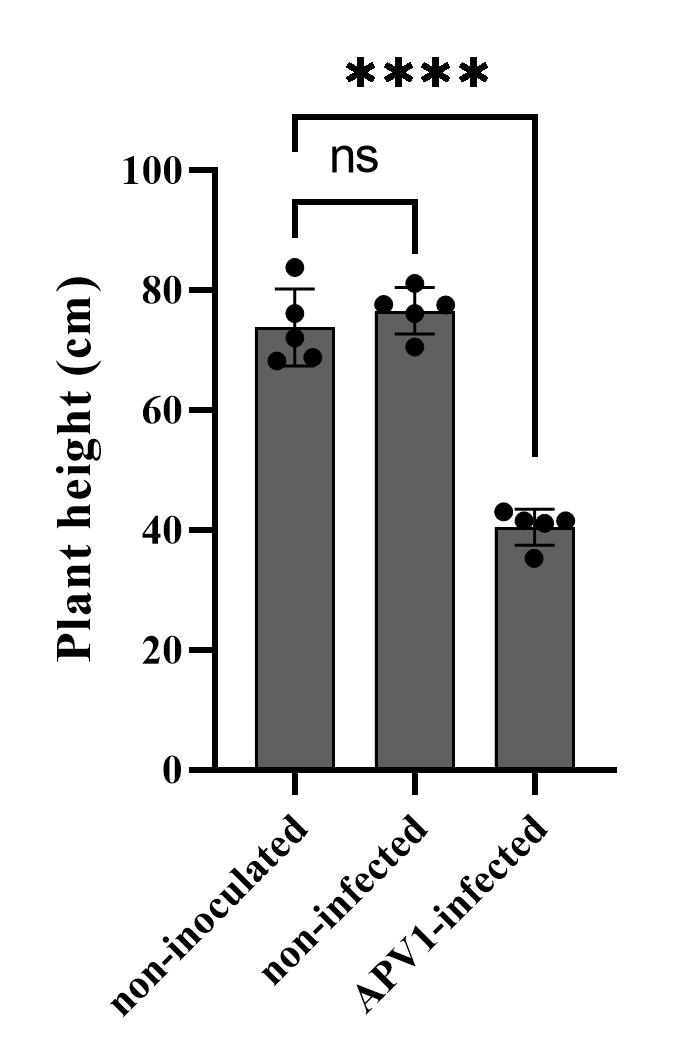
**

**Figure S1. APV1 infection caused significant growth retardation of betel palm seedlings.** Areca palm seedlings were inoculated with *Ferrisia virgata* carrying APV1 virus. The seedlings height was measured at 3 months after of inoculation. The uninoculated seedlings and non-APV1 infected seedlings after inoculation were used as the control, and 5 replicates were performed for each treatment.

**Figure S2. Volcanoplot showing deferentially expressed genes (DEG) between the APV1 infected sample (IS) and mock control of areca palm.**

**
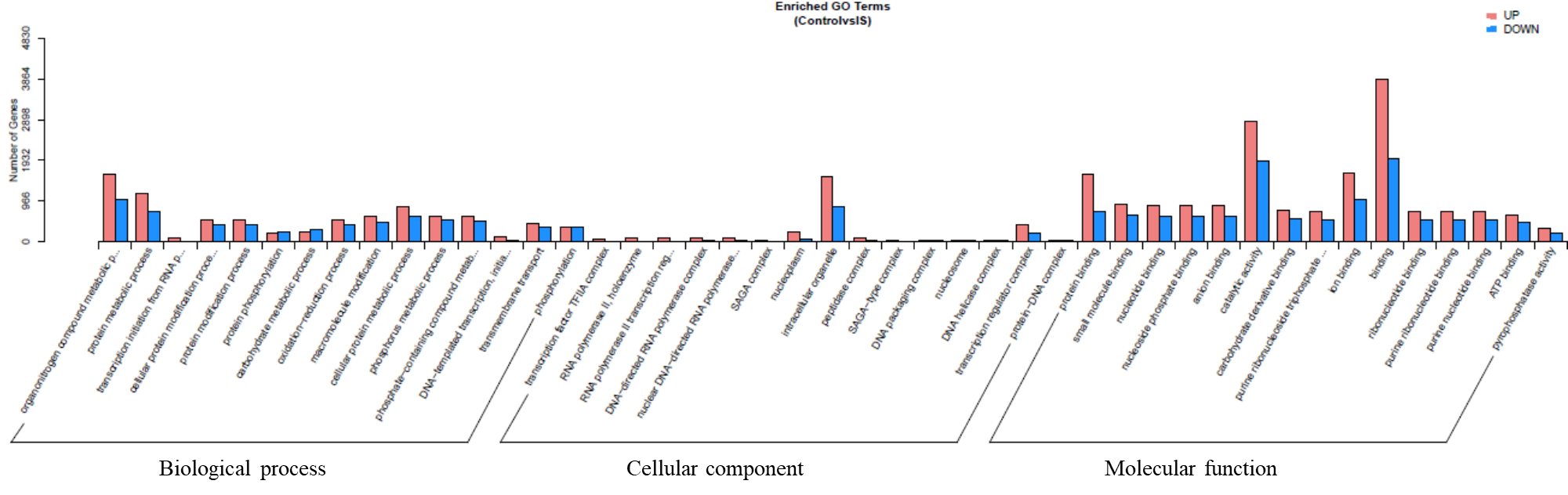
**

**Figure S3. GO enrichment of the differentially expressed genes (DEG) between the APV1 infected sample (IS) and mock control of areca palm.**

**A**


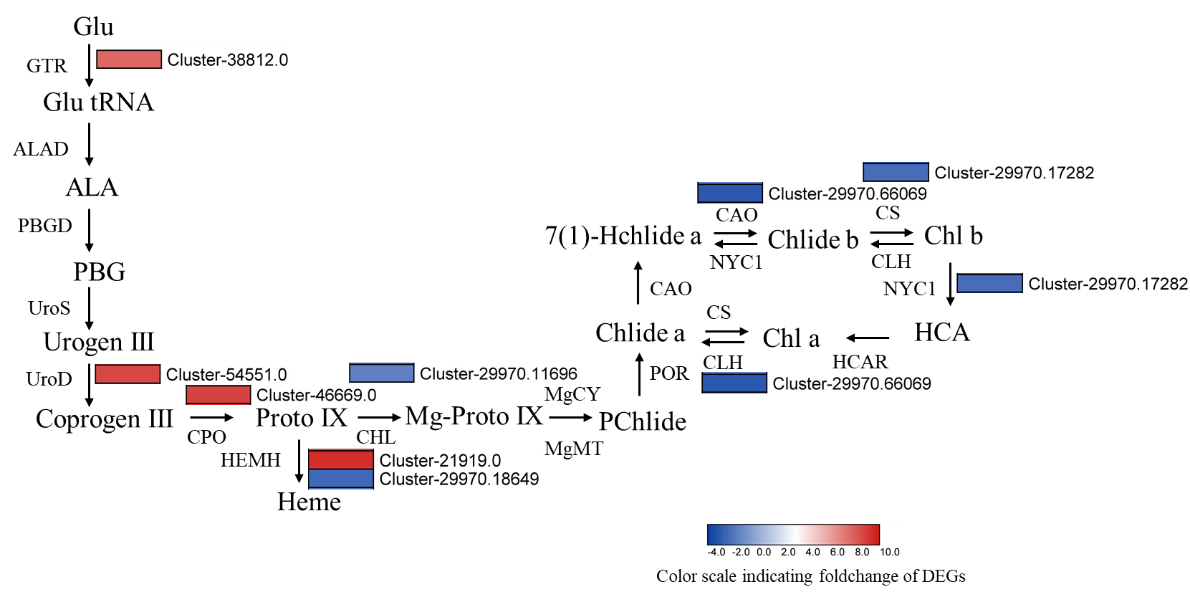

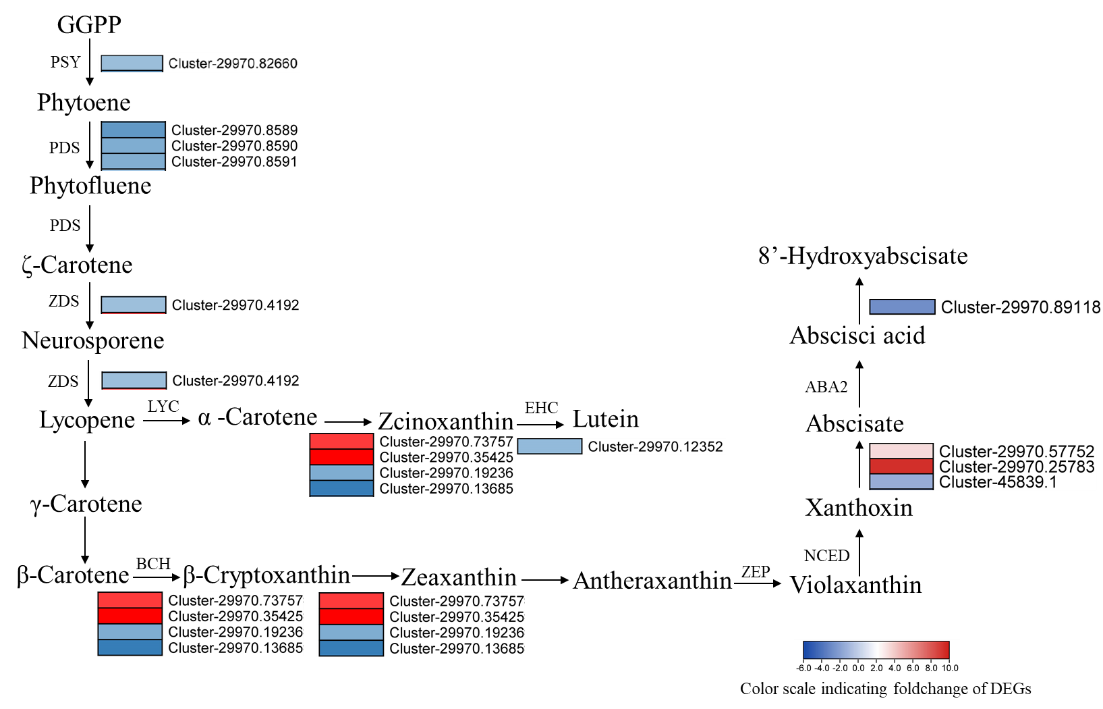


B

**Figure S4. Expression profiles of differentially expressed genes (DEGs) associated with pigment biosynthesis after APV1 infection. A.** DEGs involved in chlorophyll biosynthesis; **B.** DEGs involved in carotenoid and abscisic acid biosynthesis. Red box indicates the up-regulated genes and blue box shows the down-regulated genes after APV1 infection.

**Table S1. PCR primers used in this work.**

| Primer name | Sequence | Product size |
| --- | --- | --- |
| ZDS-F | GCTTAGGGCTTCGCTTCT | 131bp |
| ZDS-R | CGCCAACGCTTTGAGGA |  |
| PSY-F | GGCTTATGATCGCTGTG | 126bp |
| PSY-R | TGGTTCCTTATTCCGTAG |  |
| NYC1-F | TTTGAGTGGTTGTAGGGAC | 122bp |
| NYC1-R | TCTGCTGTTTTACTTAGCC |  |
| CS-F | TCACTGGACTGTGGAAGA | 140bp |
| CS-R | GAATAGGACGGTAAGGCT |  |
| AcActin-F | CCCCAGAAGAACACCCA | 119bp |
| AcActin-R | AATAGCCACATACATAGCAG |  |
